# Clinical Practice Patterns of Orthoptists in Post-Stroke Vision Care in Australia

**DOI:** 10.22599/bioj.580

**Published:** 2026-10-26

**Authors:** Konstandina Koklanis, Meri Vukicevic, Marc Hseun-Qi Eng

**Affiliations:** 1 Discipline of Orthoptics, La Trobe University, AU

**Keywords:** stroke, vision impairment, vision rehabilitation, orthoptist, care pathways, practice patterns

## Abstract

**Introduction:**

Vision impairment is common after stroke and whilst orthoptists are well placed to assess and manage post-stroke visual deficits, their role within Australian stroke care pathways is poorly understood. This study examined orthoptic practice patterns and perceived barriers to post-stroke vision care in Australia.

**Methods:**

A cross-sectional online survey of practising Australian orthoptists working in stroke care was conducted, collecting data on demographics, practice settings, referral pathways, assessment and management practices, and perceived barriers. Quantitative data were analysed descriptively, and open-ended responses thematically.

**Results:**

Twenty-nine orthoptists completed the survey, representing an estimated 22% of the orthoptic workforce engaged in stroke care. Respondents were mainly experienced clinicians working in private ophthalmology or hospital outpatient eye clinics, with limited involvement in stroke units or rehabilitation settings. Orthoptists reported managing a broad range of post-stroke visual deficits, reflected in comprehensive visual assessments and rehabilitation strategies. Orthoptic involvement most often commenced months post-stroke and key barriers to orthoptic services included limited awareness of orthoptic roles, exclusion from multidisciplinary stroke teams, funding and lack of guideline inclusion.

**Conclusion:**

Post-stroke vision care in Australia broadly aligns with international practice, but survey findings suggest that the timing and context of orthoptic involvement may be influenced by structural and organisational factors.

## Introduction

Stroke is a major public health burden in Australia, contributing substantially to both mortality and long-term disability (AIHW, 2018). Visual dysfunction is a prevalent but frequently overlooked sequela of stroke with 60% of survivors experiencing some form of vision impairment (Rowe *et al*., 2019). These visual deficits can significantly hinder recovery and independence, exacerbating psychosocial outcomes such as depression and social withdrawal (Chia *et al*., 2004; Falkenberg *et al.*, 2020). Post-stroke vision impairment encompasses a range of deficits, including disturbances of central and peripheral vision, ocular motility disorders, and higher-order visual perceptual impairments. In practice, these disturbances may present as diplopia, blurred or reduced vision, reading difficulties, and impaired recognition of people or objects, with meaningful consequences for daily functioning (Rowe, 2013b; Rowe *et al*., 2009).

While complete recovery of post-stroke visual impairment is not often possible, vision rehabilitation has been shown to effectively reduce symptom burden and functional limitations (Aimola *et al*., 2014; Pollock *et al.*, 2011a; Pollock *et al.*, 2011c; Pollock *et al.*, 2019; Rowe *et al*., 2013). Delivered through a combination of compensatory techniques, adaptive supports, and targeted interventions, vision rehabilitation can assist stroke survivors to better navigate daily activities. These approaches are central to reducing disability and promoting independence in post-stroke vision care.

International stroke guidelines increasingly recommend routine vision screening and assessment for stroke survivors as a standard component of post-stroke care (HSFC, 2025; ISWP, 2023; NICE, 2023; NDH, 2017; Rowe *et al*., 2025; SF, 2025). Within this broader international consensus, a subset of guidelines goes further by explicitly recognising orthoptists as key providers of specialist vision assessment, supported by structured referral pathways for management and follow-up. Among these, the United Kingdom and Norwegian national stroke guidelines and the European Stroke Organisation vision guidelines formally identify orthoptists and specialist eye care teams as integral to the assessment and management of post-stroke visual impairment (ISWP, 2023; NDH, 2017; Rowe *et al*., 2025). This international recognition is underpinned by a substantive evidence base demonstrating the prevalence, clinical impact, and management of post-stroke visual impairment, including the feasibility and effectiveness of orthoptic-led screening and assessment (Hanna *et al.*, 2017; Hepworth and Rowe, 2026; Pollock *et al*., 2011a; Rowe, 2017b; Rowe *et al*., 2022).

In contrast, Australian stroke care lacks explicit guidance regarding specialist vision services or orthoptists. Within the Australian and New Zealand Living Clinical Guidelines for Stroke Management, post-stroke vision care is limited to screening for visual acuity, visual field loss, and eye movement disorders, without explicit guidance on specialist vision assessment, orthoptic involvement, or structured post-stroke vision rehabilitation pathways (SF, 2025). Available evidence indicates that post-stroke vision care pathways in Australia are largely unstandardised, with substantial variability in who undertakes vision assessment and management, when this occurs, and how referrals are made across care settings (Sorbello *et al*., 2024). While orthoptists are known to be engaged in the assessment and management of visual symptoms following stroke, including the provision of targeted interventions within hospital settings (Macfarlane *et al*., 2011), there is limited contemporary Australian evidence describing their role within stroke teams or how this involvement is integrated within broader care pathways. The aim of this study was to evaluate current orthoptic practice patterns and referral pathways in post-stroke vision care in Australia, including practice settings and perceived barriers to service delivery.

## Materials and Methods

### Study design

A cross-sectional study was conducted surveying practising orthoptists in Australia. Ethics approval was obtained from the La Trobe University Human Research Ethics Committee.

### Participants

Orthoptists working in clinical practice in Australia were eligible to participate. There were no restrictions based on clinical setting, level of experience in post-stroke vision care, or frequency of stroke patients encountered. Participants were invited to take part via an email distributed to members of Orthoptics Australia, the national professional association for orthoptists, and through promotion on professional social media platforms (LinkedIn). The invitation contained a secure link to the online survey, which opened with an overview of the study and the participant information and consent form prior to survey commencement.

### Survey

Data were collected using a purpose-designed online survey administered via the Research Electronic Data Capture (REDCap) platform, with responses collected in a de-identified manner. The survey comprised of 37 closed-choice items and one open-ended question, including 13 items employing branching logic. The questions focused on five areas: participant demographics, practice settings, clinical practice patterns, referral pathways, and perceived barriers to care. The survey was informed by previous international research examining the role of orthoptists in post-stroke vision care (Rowe, 2017a) and was reviewed by an Australian orthoptist prior to dissemination to ensure clinical relevance and clarity. Estimated completion time was up to 20 minutes and the survey remained open for six weeks.

The full survey instrument is provided as a Supplementary File.

### Data analysis

Survey data stored in REDCap were exported to SPSS (IBM Corp., Version 29) for quantitative analysis of closed-response items. Descriptive statistics were generated for participant characteristics, including frequencies and percentages for categorical variables. Open-ended survey responses were analysed using a thematic analysis approach. Responses were reviewed in full and coded inductively to identify recurring issues and topics raised by respondents.

## Results

Surveys were completed by 29 participants, with a further nine individuals providing consent but not completing the survey. Of the 29 submitted surveys, 21 were fully completed and eight contained missing responses for one or more items. Incomplete responses were retained for analysis, with item-level denominators reported where applicable.

The response rate is estimated to be approximately 22%, based on publicly available registration data indicating that there were 875 registered orthoptists in Australia at the time of the survey (AOB, 2025) and workforce data suggesting that approximately 15% of orthoptists are involved in stroke care (OA, 2024).

### Participants

Of the 29 orthoptists who participated in the study, most respondents were aged 41 years or older (n = 20, 68.9%), while nine participants (31.0%) were under 40 years of age, including three under 30 years. Most orthoptists had extensive professional experience, with 18 participants (62.1%) reporting more than 20 years of practice. Five respondents (17.2%) had between 10 and 20 years of experience, and six (20.6%) had 10 years or less. The cohort was predominantly female (n = 22, 75.9%), with seven male participants (24.1%). Experience working specifically in stroke care was reported by 14 participants (48.3%) as more than 20 years, by seven (24.1%) as between 10 and 20 years, and by eight (27.6%) as 10 years or less. Respondents were geographically distributed across Australia, with equal representation from Victoria and New South Wales (n = 11, 37.9% each), followed by Queensland (n = 4, 13.8%), South Australia (n = 2, 6.9%), and Western Australia (n = 1, 3.4%).

### Workplace characteristics

Most respondents reported working in metropolitan areas (22, 75.9%), with the remainder working in regional or rural settings (7, 24.1%). Employment settings varied, with respondents working in public (10, 29.4%), private (18, 52.9%), and non-government or not-for-profit organisations (6, 17.6%), with five respondents reporting work across multiple organisation types. Clinical practice settings were most commonly private ophthalmology practice (14, 36.8%) and hospital outpatient eye clinics (8, 21.1%), followed by hospital acute inpatient wards (4, 10.5%), private orthoptic practice (4, 10.5%), and low-vision organisations (4, 10.5%). Smaller numbers reported working in subacute inpatient rehabilitation facilities (2, 5.3%), community rehabilitation facilities (1, 2.6%), or residential care settings (1, 2.6%).

Five respondents (17.2%) reported working in facilities that included a stroke unit, however, all indicated that they did not work directly within the stroke unit. Among these respondents, four reported being unsure which discipline assessed vision within the stroke unit, while one reported that assessment was undertaken by the on-call ophthalmology team or through inpatient referral to the outpatient eye clinic.

### Referral patterns to orthoptists

Respondents reported receiving referrals from a range of health professionals, most commonly ophthalmologists (19, 18.4%), optometrists (16, 15.5%), and neurologists (14, 13.6%). Allied health referral sources reported by respondents included occupational therapists (10, 9.7%), physiotherapists (9, 8.7%), and speech pathologists (6, 5.8%), with additional referral sources including stroke consultants (6, 5.8%), general practitioners (7, 6.7%), and orthoptists (5, 4.9%). Self-referral was reported as a referral source by eight respondents (7.8%). Less commonly reported referral sources included paediatricians, psychologists, and low-vision organisations (n = 1, 0.98% each).

Respondents also reported a range of referral requests reflecting both diagnostic and rehabilitative needs. Diagnostic-focused requests included specific clinical investigations (23, 22.5%), comprehensive vision assessment (23, 22.5%), post-stroke vision screening (18, 17.6%), or assessment for a specific diagnosis (15, 14.7%). Requests for management and vision rehabilitation were reported by 22 respondents (21.6%). One respondent (1%) reported receiving a referral requesting a driving assessment.

Where referrals specified requests for a clinical investigation, visual field assessment was most commonly reported (23, 28.4%), followed by ocular motility examination (20, 24.7%) and visual acuity assessment (19, 23.5%). Requests for refraction were reported by nine respondents (11.1%), and an assessment of visual perception, including visual neglect, was reported by eight respondents (9.9%). A small number of respondents selected ‘other’ investigations (2, 2.5%), which included requests for diplopia assessment, pupil assessment, fundus photography and optical coherence tomography.

### Presenting symptoms and visual deficits

Respondents reported a wide range of visual symptoms among patients presenting following stroke. The most frequently reported presenting symptoms were loss of peripheral vision or parts of vision (21, 91.3%), diplopia (21, 91.3%), and bumping into objects or poor spatial awareness (21, 91.3%). Reading difficulty was also common (20, 87.0%), followed by blurred vision (19, 82.6%). Depth perception difficulties were reported by over half of respondents (12, 52.2%). A range of additional symptoms were reported less frequently, including recognition difficulties (9, 39.1%), facial droop (9, 39.1%), shaky vision (8, 34.8%), abnormal head posture (8, 34.8%), writing difficulty (8, 34.8%), lid droop (8, 34.8%), and photophobia (8, 34.8%). Colour vision changes were reported by four respondents (17.4%), and one respondent (4.3%) reported ‘other’ symptoms which included watery eyes. The range of post-stroke visual deficits reported by respondents is illustrated in Figure 1.

**Figure 1:**
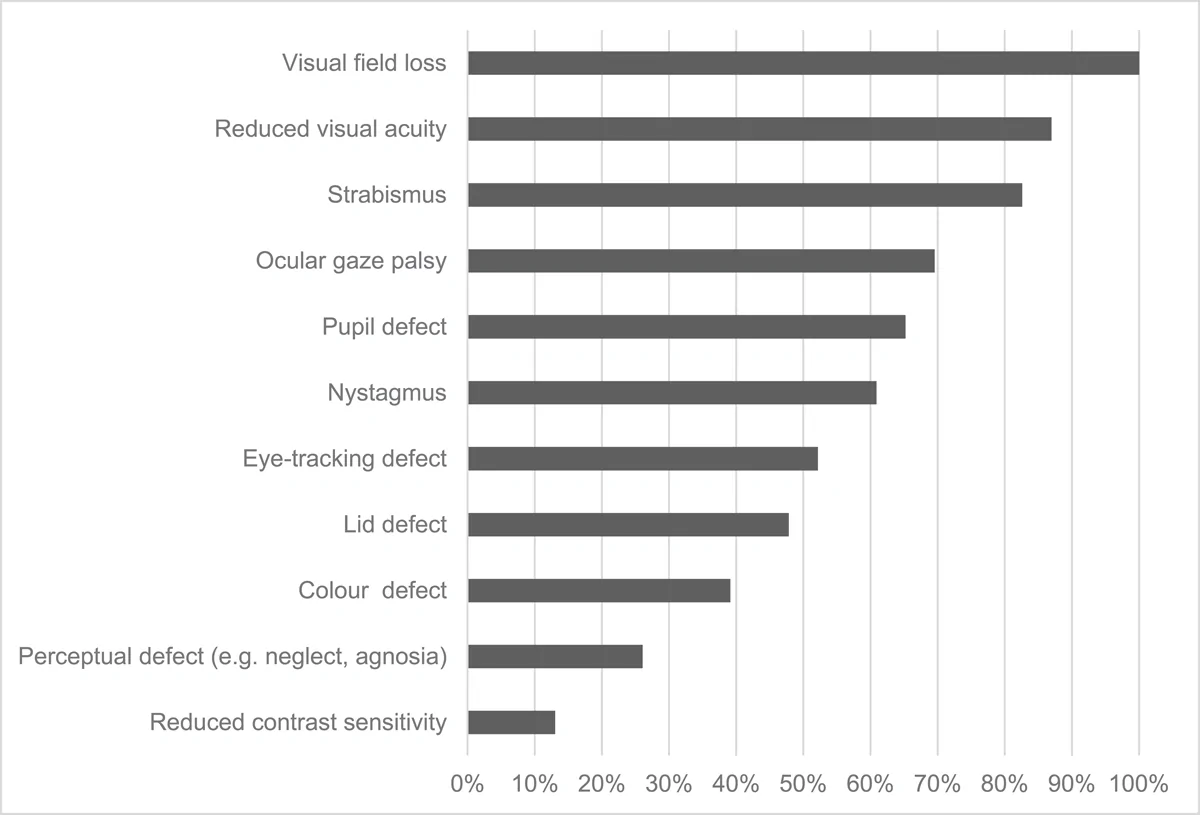
Frequency of post-stroke visual deficits identified in orthoptic practice.

### Timing of initial post-stroke vision assessment

Individual respondents reported multiple common time points for initial post-stroke vision assessment, resulting in more responses than respondents. Respondents most commonly reported first seeing patients between 3–6 months (10, 20%) or more than 6 months (10, 20%) post-stroke, with fewer reporting initial assessment at 1–2 months (9, 18%), 2–4 weeks (8, 16%), within two weeks (2, 4%), or within one week (2, 4%) following stroke.

### Clinical assessment

The range and frequency of clinical assessments reported by respondents are shown in Figure 2, with visual acuity and visual field assessment reported by all respondents as the most commonly performed assessments. Two respondents selected ‘other’, indicating optical coherence tomography (n = 1) and assessments undertaken as required or requested (n = 1). Table 1 summarises the assessment methods used.

**Figure 2:**
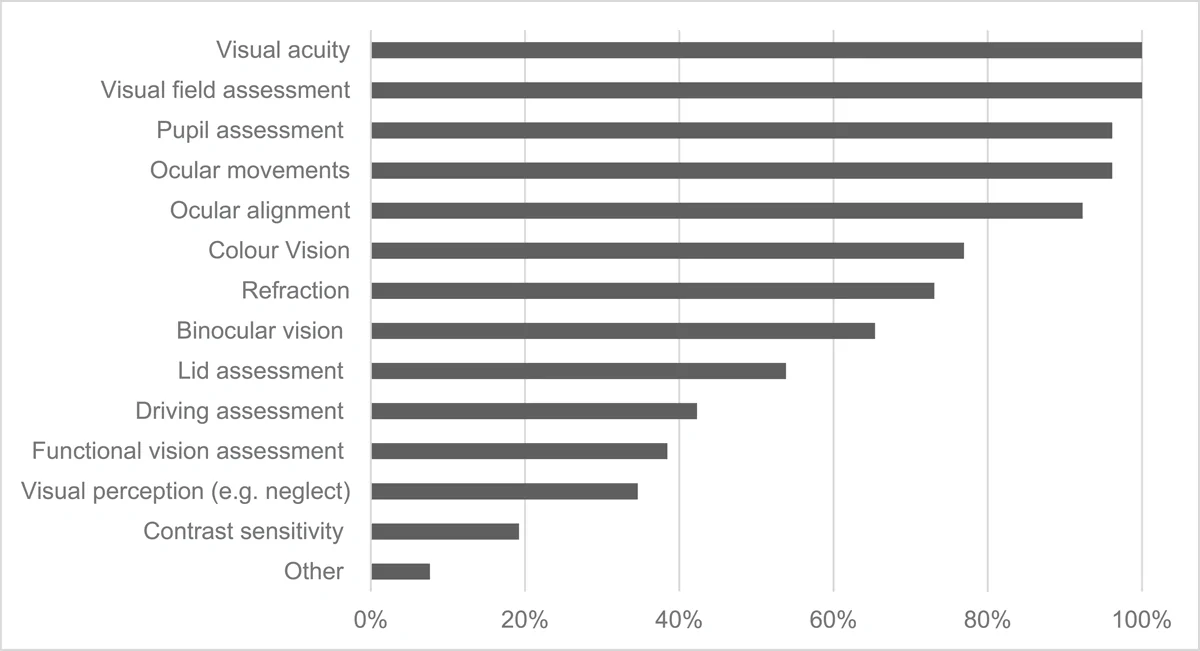
Frequency of vision assessments performed by orthoptists following stroke.

**Table 1:** Visual assessment components performed by respondents.


ASSESSMENT DOMAIN	ASSESSMENT METHOD

**Visual acuity**	Near visual acuity (26.2%) Snellen chart (24.6%) LogMAR chart (21.5%) Fixing and following CSM (13.8%) Grading acuity (9.2%) Other: Dynamic visual acuity, letter matching (4.6%)

**Refraction**	Subjective refraction (68.0%) Objective refraction (24.0%) Other: Auto-refraction, Corneal topography (8.0%)

**Ocular movements**	Cover tests (15.9%) Nine positions of gaze (15.9%) Saccades (15.2%) Convergence (14.6%) Smooth pursuit (14.6%) Assessment of corneal reflections (9.9%) Hess chart (4.6%) Vestibulo-ocular reflex (4.0%) Optokinetic nystagmus (OKN) (3.3%) Other: prism cover test (1.3%) Other: field of BSV (0.7%)

**Binocular vision**	Lang I and II (19.4%) Prism fusion range (16.1%) Titmus stereotests (16.1%) TNO stereotests (11.3%) Worth 4 dot tests (11.3%) 20Δ prism fusion tests (9.7%) Synoptophore investigation (6.5%) Frisby Davis stereotests (4.8%) Bagolini striated glasses tests (4.8%)

**Visual fields**	Automated perimetry (40.4%) Confrontation (34.6%) Goldmann perimetry (11.5%) Bjerrum tangent screen (9.6%) Other: Lister kinetic perimetry, arc perimetry (3.8%)

**Colour vision tests**	Ishihara (69.2%) Farnsworth–Munsell D-15 (19.2%) CUVCT (7.7%) Other: pseudo-isochromatic test, colour matching test (2.6%)

**Contrast sensitivity**	Vistech (33.3%) Pelli–Robson (16.7%) Melbourne Edge Test (16.7%) Other: Mars contrast test (16.7%)

**Visual perception**	Drawing (25.0%) Line bisection tests (20.0%) Cancellation tests (20.0%) Copying (20.0%) Other: Extinction testing, Boston Cookie Test, Motor-Free Visual Perception Test (MVPT) (15.0%)

**Functional vision tests**	Observation of function/navigation (20.5%) Reading (17.9%) Activities of daily living (17.9%) Mobility (17.9%) Writing (15.4%) Eye scanning (5.1%) Locally developed visual function questionnaire (2.6%) Validated visual functional questionnaire (0%)


### Management strategies

Figure 3 illustrates the range of management and rehabilitation strategies prescribed by respondents. The most commonly reported strategies included prisms for diplopia (17, 73.9%), optical correction, monocular patching for diplopia, and orthoptic exercises (15, 65.2% each), followed by vision-related advice (13, 56.5%) and visual scanning for visual field loss (12, 52.2%).

**Figure 3:**
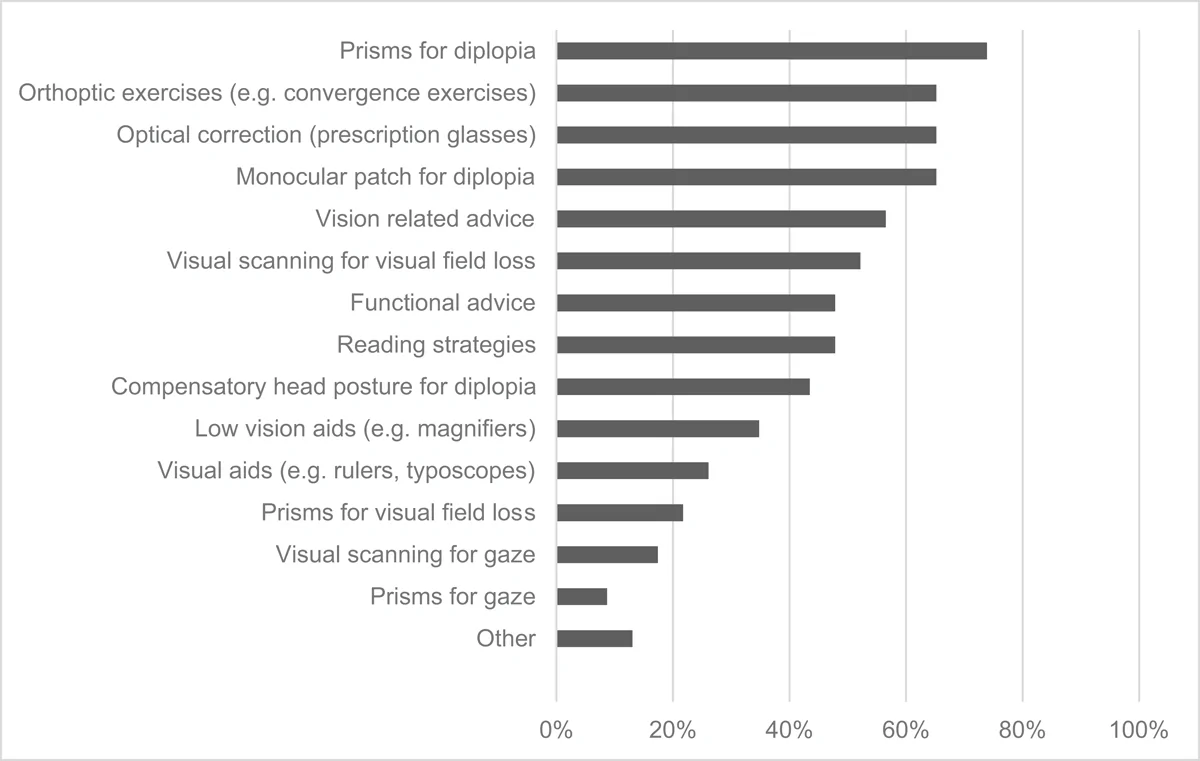
Management strategies employed by orthoptists in post-stroke vision care.

Respondents reported considerable variation in the timing of discharge or cessation of follow-up for patients with post-stroke vision difficulties. Ongoing review was common, with 10 respondents (43.5%) reporting that patients were reviewed indefinitely. Longer-term follow-up was also frequently reported, with discharge occurring after 12 months in eight respondents (34.8%) and between 9–12 months in four respondents (17.4%). Shorter follow-up periods were less commonly reported, with discharge within 2–3 months and 3–6 months each reported by three respondents (13.0%), and within 6–9 months by two respondents (8.7%).

### Multidisciplinary collaboration in post-stroke vision rehabilitation

Of 23 respondents, 14 (60.9%) reported collaborating with other health professionals in the delivery of post-stroke rehabilitation services, while nine respondents (39.1%) reported that they were not engaged in any multidisciplinary care. Among those who reported multidisciplinary involvement, collaboration was most commonly reported with ophthalmologists (11, 78.6%), neurologists (9, 64.3%), occupational therapists (8, 57.1%), and optometrists (8, 57.1%). Physiotherapists were also frequently involved (7, 50.0%), followed by speech pathologists (6, 42.9%). Less commonly reported collaborators included psychologists (4, 28.6%) and social workers (3, 21.4%). A small number of respondents also reported collaboration with ‘other’ professionals (4 respondents, 28.6%). These responses included orientation and mobility specialists (n = 2), optometrists (n = 1), optical dispensers (n = 1), neuropsychologists (n = 1) and practitioners in Vision Australia (n = 1).

### Perceived barriers to post-stroke orthoptic care

Figure 4 summarises the most commonly reported barriers to post-stroke orthoptic care identified by 21 respondents, with lack of knowledge regarding the role of the orthoptist in stroke care being the most frequently reported barrier.

**Figure 4:**
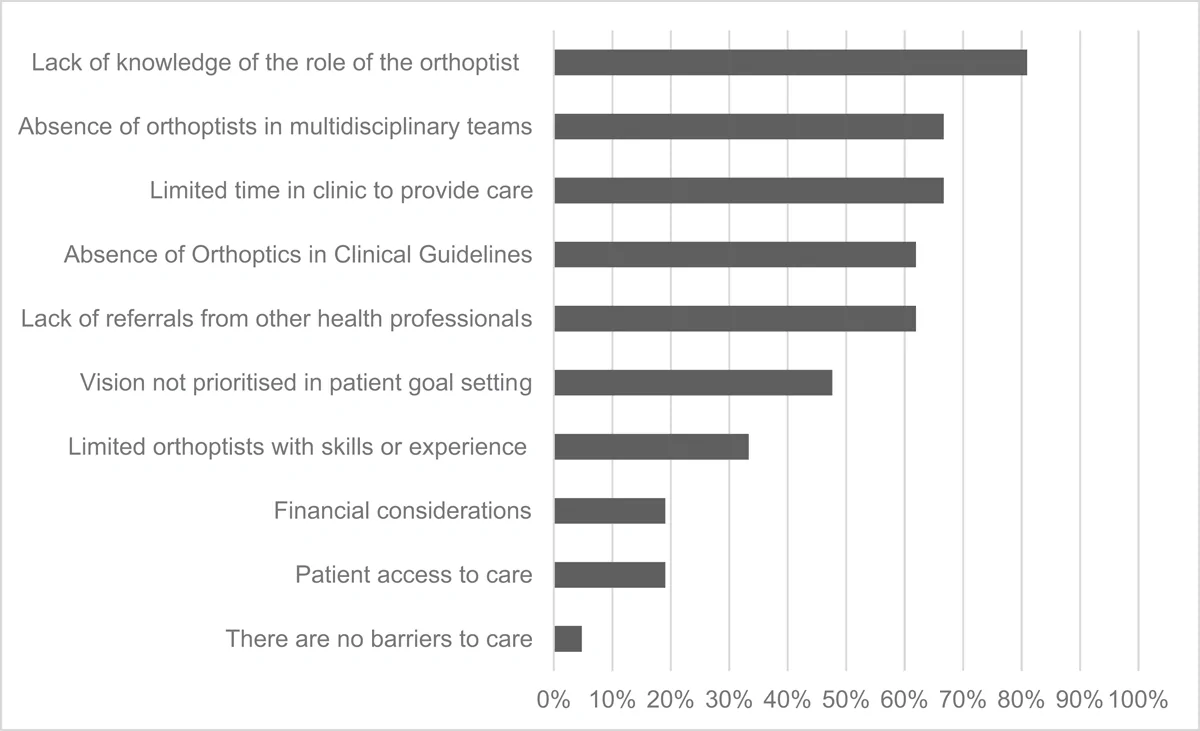
Frequency of perceived barriers to post-stroke orthoptic care.

### Qualitative findings: perceived enablers and service improvement opportunities

Seven respondents provided open-ended comments regarding enablers, gaps, and opportunities related to post-stroke orthoptic services. A recurring issue raised was the need for inclusion of orthoptists within stroke care teams, including acute stroke services and multidisciplinary stroke meetings. In addition, respondents noted that orthoptists should be involved beyond clinic-based assessment, including participation in patient home visits, particularly where patients experience mobility or financial barriers to attending services.

Respondents also highlighted system-level factors affecting service provision. Concerns were raised regarding the absence of recognition of orthoptists in stroke care, limited funding for orthoptic positions within stroke services, and lack of inclusion of orthoptics within stroke guidelines and protocols. Some comments described constraints within public health settings, including appointment structures and funding models that prioritise outpatient throughput.

A further theme related to education, awareness, and referral pathways. Limited awareness of the orthoptic role among medical and allied health professionals was described, with respondents noting that referrals for post-stroke vision care were dependent on service awareness. Suggested enablers included clearer orthoptic-specific referral pathways, increased professional education and continuing development in post-stroke vision care, greater collaboration with hospital and community medical services, and advocacy at professional and association levels.

## Discussion

This study represents the first Australian investigation to specifically examine orthoptic practice patterns in post-stroke vision care. The findings provide novel insight into workforce characteristics, service delivery models, referral pathways, and perceived barriers to orthoptic involvement in stroke rehabilitation, and highlight several structural and system-level issues that warrant further exploration.

It was noteworthy that the orthoptic workforce represented in this study was predominantly older and highly experienced, raising potential concerns regarding future workforce sustainability as senior clinicians approach retirement. This age distribution appears inconsistent with previously reported national workforce data. In the most recently published Survey reported by Jolly *et al*. (2019), approximately half of respondents were under 40 years of age, with only 13.2% aged 60 years or older. In contrast, the current sample was heavily weighted toward clinicians aged 41 years and over, with a substantial proportion reporting more than 20 years of professional experience. However, given the small sample size and potential response bias, these findings should be interpreted cautiously. The age profile observed may reflect the characteristics of orthoptists who responded to this survey, rather than the broader orthoptic workforce or all orthoptists involved in post-stroke vision care. Further workforce research is needed to determine whether post-stroke vision care is concentrated among more experienced orthoptists and to consider the implications for workforce planning and service sustainability.

The older age profile observed in this study may also reflect the advanced clinical skills and professional autonomy often required to manage complex neurological presentations. However, it is also possible that early-career orthoptists may have fewer opportunities to work in stroke care due to limited funded positions or the lack of formal integration of orthoptists within multidisciplinary stroke teams. In this context, attrition among this senior cohort may constrain access to specialised post-stroke vision services over time, with potential implications for service continuity, clinical mentorship, and the maintenance of specialist expertise within stroke care. This highlights the importance of targeted workforce planning, succession strategies, and the development of supported career pathways to enable earlier engagement of orthoptists in stroke rehabilitation services and to support the long-term sustainability of care.

The workplace characteristics identified in this study indicate that orthoptists most commonly encounter post-stroke patients within private ophthalmology practices or hospital outpatient eye clinics, with limited representation of orthoptists in community rehabilitation, inpatient rehabilitation, and stroke unit settings. This pattern is consistent with broader descriptions of post-stroke vision care as largely unstandardised, with gaps relating to the integration of eye-care specialists into the stroke team (Sorbello *et al*., 2024). In private ophthalmology practice settings in Australia, orthoptists typically work closely with ophthalmologists but have limited routine interaction with other health professionals involved in stroke rehabilitation. Similarly, within hospital environments, orthoptists are frequently positioned within medical or ophthalmology divisions rather than allied health services. While these service models support comprehensive visual assessment and the medical or episodic management of visual conditions, they contribute to siloed models of care that are less well suited to the delivery of rehabilitation-focused vision care. This interpretation is supported by the finding that almost 40% of respondents in the present study reported no collaboration with other health professionals in the delivery of post-stroke rehabilitation services. Collectively, these factors suggest that access to specialist orthoptic input may be constrained for many stroke survivors, particularly during the acute and subacute phases following stroke.

Referral patterns further reflect this service configuration. Most referrals originated from medical practitioners and optometrists, with fewer referrals from allied health professionals. This likely reflects both the predominance of orthoptists working within ophthalmology-based settings and limited awareness of the orthoptic role in post-stroke vision care among some disciplines. Gaps in awareness, knowledge, and training related to post-stroke vision difficulties have been reported in previous studies and identified as a barrier to access to appropriate post-stroke vision care (Hanna *et al*., 2020; Pollock *et al*., 2011b; Rowe *et al*., 2015; Sorbello *et al*., 2024). This again may result in delayed or inconsistent access to orthoptic assessment and vision rehabilitation following stroke.

The presenting visual symptoms identified in this study align closely with international reports, including visual field loss, diplopia, spatial awareness difficulties, reading difficulty, and blurred vision (Rowe, 2013b; Rowe, 2017a). Correspondingly, the clinical assessments reported demonstrate relative alignment with international orthoptic practice, with high uptake of core assessments such as the evaluation of visual acuity, visual fields, ocular motility, and binocular vision alongside the use of validated testing instruments (Rowe, 2017a; Rowe and VIS Writing Group, 2017), suggesting consensus regarding key post-stroke visual sequelae and essential assessment components.

Management strategies reported by respondents in this study closely aligned with those described in Rowe’s international survey of orthoptists (Rowe, 2017a), reflecting a rehabilitation-oriented approach to post-stroke vision care. Interventions such as prisms, optical correction, occlusion, orthoptic exercises, visual scanning strategies, and tailored vision-related advice are typically directed toward reducing activity limitations affecting tasks such as reading, mobility, and environmental navigation, while also supporting safer mobility and participation in rehabilitation and everyday life (Hanna *et al*., 2017).

However, notable differences were observed in the timing of initial orthoptic assessment and follow-up practices. Orthoptists in this study most commonly reported first seeing patients several months after stroke, with relatively few patients assessed during the acute or early subacute phases. This pattern likely reflects the limited presence of orthoptists within acute stroke units and inpatient rehabilitation services. In contrast, international data reported by Rowe (2017a) indicate substantially earlier orthoptic involvement, with over one-third of orthoptists seeing patients within two weeks of stroke and more than half within the first month. This contrast suggests that service configuration and workforce integration may play a key role in determining the timeliness of post-stroke vision assessment.

Differences were also observed in follow-up duration. In Rowe’s international survey (Rowe, 2017a), follow-up was frequently reported as less than 3 months for many patients, whereas respondents in the present study commonly described longer-term or ongoing review, with a substantial proportion following patients for 9 to 12 months or indefinitely. Although direct comparison is limited due to the absence of condition-specific follow-up analysis in our study, this may reflect later access to orthoptic care in Australia, potentially necessitating prolonged follow-up to address more established or unmet rehabilitation needs. Longer follow-up could also reflect differences in case mix, outpatient service models, funding structures, or clinician discharge practices and is worth further exploration.

Alongside the practice patterns described, this study identified multiple barriers to the provision of post-stroke orthoptic care. The most frequently reported barriers were limited recognition of the orthoptic role, lack of inclusion within multidisciplinary stroke teams, and insufficient time within clinical settings to support stroke care. Qualitative responses reinforced these findings and further highlighted broader system-level limitations, including insufficient funding and the absence of orthoptists within formal stroke guidelines.

While limited awareness of post-stroke visual impairment has been widely reported in the literature (Hanna *et al*., 2020; Pollock *et al*., 2011b; Rowe, 2013a; Sorbello *et al*., 2024), respondents in the present study identified limited recognition of the orthoptic role itself as a distinct barrier, extending beyond the general awareness of post-stroke visual impairment. This lack of role recognition is likely associated with limited inclusion of orthoptists within multidisciplinary stroke teams and greater integration within ophthalmology or medically led services, potentially reducing role visibility and shared understanding through routine interdisciplinary practice. Time constraints were also commonly reported and likely reflect prevailing service models in which orthoptists work, which are typically structured for episodic assessment and management rather than rehabilitation-focused care.

Consistent with previous research, lack of funding emerged as a barrier to service provision (Hepworth and Rowe, 2026; Pollock *et al*., 2011a; Rowe *et al*., 2016; Rowe, 2017a; Sorbello *et al*., 2024). Indeed, in the present study, all respondents working within hospitals that included a stroke unit reported that they did not provide care directly within the unit, indicating an absence of orthoptic involvement in the acute phase of stroke care. In addition, few respondents reported working within community-based rehabilitation settings, suggesting limited orthoptic involvement across both acute and post-acute stages of the stroke care pathway.

In the Australian context, the absence of orthoptists from stroke clinical guidelines may further perpetuate these challenges. Internationally, there is growing consensus that vision assessment and rehabilitation should form a routine component of post-stroke care, with multiple stroke guidelines recommending vision screening, specialist assessment, and/or the management of post-stroke visual impairment for stroke survivors (HSFC, 2025; ISWP, 2023; NDH, 2017; Rowe *et al*., 2025; SF, 2025). This includes the Australian and New Zealand Living Clinical Guidelines for Stroke Management, which recommend assessment of visual acuity, visual field deficits, and eye movement disorders for all stroke survivors (SF, 2025). However, within the Australian guidelines, vision-related recommendations are largely embedded within occupational therapy practice and do not extend to recommending specialist eye care involvement or identifying orthoptists as providers within the stroke team. In contrast, a number of international guidelines more explicitly recognise orthoptists as members of specialist eye care teams and recommend their involvement in the delivery of post-stroke vision assessment and management. Additionally, evidence from UK service evaluations suggests that this recognition of orthoptists within guidelines has been associated with increased provision of vision services and greater access to orthoptic care within stroke units over time (Hepworth and Rowe, 2026). The extent to which orthoptists are integrated into Australian stroke services, and the impact of their absence from national guidelines, remains largely unevaluated.

### Limitations

This study has several limitations that should be considered when interpreting the findings. The sample size was relatively small, reflecting the specialised workforce of orthoptists involved in post-stroke vision care in Australia. The response rate was estimated to be approximately 22% based on available registration and workforce data; however, both this estimate and the generalisability of the findings should be interpreted with caution, as the presence of nonresponse bias cannot be excluded.

In addition, the use of a self-reported survey may have limited the depth and nuance of information obtained. Responses reflect participants’ interpretations of the survey items and may not fully capture the complexity of local practice, service arrangements, or clinical decision-making. Missing data and variability in responses may reflect differences in terminology or service models across settings.

The findings of this study are also limited to the professional perspective of orthoptists and do not capture the views of other stakeholders involved in post-stroke vision care, including other members of the multidisciplinary stroke team, patients, and carers. Future studies incorporating health service mapping, multidisciplinary perspectives, and lived experience perspectives from stroke survivors and carers are needed to better characterise how post-stroke vision care is accessed and coordinated, including barriers to care and unmet needs. Such work would support the development of contextually appropriate guidance and address gaps in the limited Australian evidence base, which is currently informed largely by UK services (Sorbello *et al*., 2024).

## Conclusion

This study provides the first national insight into orthoptic practice in post-stroke vision care in Australia, identifying workforce, service delivery, and system-level factors that are associated with access to orthoptic expertise. Although assessment and management approaches reported by respondents are broadly consistent with international practice, orthoptic involvement commonly occurs later in the stroke care pathway and remains largely situated within outpatient and ophthalmology-based models that may be less aligned with rehabilitation-focused care pathways. Barriers to timely and integrated service delivery were more frequently attributed by respondents to structural factors rather than clinical capability, including limited role recognition, lack of inclusion of orthoptists within multidisciplinary stroke teams, funding constraints, and the absence of orthoptics within formal stroke pathways and clinical guidelines. The concentration of post-stroke vision care among senior clinicians further highlights patterns within the current workforce that may have implications for sustainability and succession. These findings underscore areas for further investigation, including care pathways, service integration, and workforce and funding arrangements, to better understand factors influencing the timing and coordination of post-stroke vision care in Australia.

## Additional File

The additional file for this article can be found as follows:

10.22599/bioj.580.s1 Supplementary File. Post-Stroke Orthoptics Vision Care Survey.
