# Supplementary material for: Clinical Practice Patterns of Orthoptists in Post-Stroke Vision Care in Australia
Source: Br Ir Orthopt J. 2026 Oct 26;22(1):150–61. doi: 10.22599/bioj.580 (PMC13722020; doi:10.22599/bioj.580)
Supplement: Supplementary file 1 [file bioj-22-1-580-s1.pdf]

Post-Stroke Orthoptics Vision Care Survey

Participant Characteristics

Which age group do you belong to?

○ < 25

○ 26-30

○ 31-35

○ 36-40

○ 41-45

○ 46-50

○ >51

How many years of experience do you have working as an orthoptist?

○ 1-3 years

○ 3-5 years

○ 6-10 years

○ 11-15 years

○ 16-20 years

○ >20 years

How many years of experience do you have providing clinical care to patients who have had a stroke?

○ 1-3 years

○ 3-5 years

○ 6-10 years

○ 11-15 years

○ 6-20 years

○ >20 years

Which of the following best describes your gender?

○ Female

○ Male

○ Non-binary

○ Other

Please specify "Other" ________________________________

Workplace and Service Characteristics

Which state or territory are you currently working in?

○ Australian Capital Territory

○ New South Wales

○ Northern Territory

○ Tasmania

○ South Australia

○ Victoria

- ○ Queensland
- ○ Western Australia

Which of the below best describes the location of your workplace?

- ○ Metropolitan
- ○ Rural
- ○ Remote

Which type of organisation(s) best describes where you provide stroke care services? (you may select more than one option)

- ☐ Public
- ☐ Private
- ☐ Non-government organisation
- ☐ Other

Please specify "Other" ____

Which of the below best describes the setting(s) in which your services are delivered? (select all that apply)

- ☐ Hospital inpatient ward
- ☐ Hospital outpatient eye clinic
- ☐ Inpatient rehabilitation facility
- ☐ Residential care facility/Rest home
- ☐ Community rehabilitation facility
- ☐ Private orthoptic practice
- ☐ Private ophthalmology practice
- ☐ Low Vision Organisation (e.g. Vision Australia, Guide Dogs)
- ☐ Other

*Please specify "Other"*____________________________________

Which professionals refer to you or your clinic for post-stroke vision care? ((you may select more than one option)

- ☐ Occupational therapist
- ☐ Physiotherapist
- ☐ Speech pathologist
- ☐ Neurologist
- ☐ Stroke consultant
- ☐ Orthoptist
- ☐ Optometrist
- ☐ Ophthalmologist
- ☐ Patient self-referral
- ☐ Other

Please specify "Other" ____

What do the referrals you receive typically request? (you may select more than one option)

- ☐ Post-stroke vision screening
- ☐ Specific investigations (e.g. visual field)
- ☐ General post-stroke vision assessment

- ☐ Diagnosis of vision related difficulty or condition
- ☐ Management of post-stroke vision difficulties
- ☐ Other

Please specify "Other" ______________________________

What specific clinical investigations are typically requested?

- ☐ Visual acuity
- ☐ Refraction
- ☐ Visual field
- ☐ Visual perception (e.g. visual neglect)
- ☐ Ocular motility examination
- ☐ Other

Please specify "Other" ______________________________

Does the hospital or facility you work in have a stroke unit?

- ○ Yes
- ○ No

Do you deliver services within the stroke unit?

- ○ Yes
- ○ No

In which specific setting do you deliver these services? (you may select more than one option)

- ☐ Hospital inpatient ward/bedside
- ☐ Inpatient rehabilitation facility
- ☐ Other

Please specify "Other" ______________________________

Who delivers vision care services in the stroke unit?

- ○ Nurse
- ○ Occupational therapist
- ○ Ophthalmologist
- ○ Neurologist
- ○ I am not sure
- ○ Other

Please specify "Other" ____________________

Are patients from the stroke unit referred to the outpatient eye clinic in the hospital or the orthoptist?

- ○ Yes
- ○ No

If you receive referrals from multiple sources, do the reasons for referral differ from the above when received from the internal stroke unit compared to other referral sources?

- ○ Yes
- ○ No

If yes, briefly explain the different type of referrals received from the stroke unit.

____

Post-stroke Clinical Assessment

How frequently do you encounter a patient(s) with post-stroke vision difficulties in your workplace?

- ○ Daily
- ○ Weekly
- ○ Monthly
- ○ Every 2-3 months
- ○ Every 4-6 months
- ○ Less than every 6 months

How frequently do you encounter a patient(s) with post-stroke vision difficulties who also have communication difficulties (e.g. aphasia)

- ○ Daily
- ○ Weekly
- ○ Monthly
- ○ Every 2-3 months
- ○ Every 4-6 months
- ○ Less than every 6 months

How soon after a stroke do you most commonly see a patient for the first time? (you may select more than one option)

- □ Within 1 week
- □ Within 2 weeks
- □ Between 2-4 weeks
- □ Between 1-2 months

- ☐ Between 2-3 months
- ☐ Between 3-6 months
- ☐ After 6 months

Do you or your clinic utilise guidelines for post-stroke vision care?

- ○ Yes
- ○ No

What guideline(s) do you utilise? ________________________________

What does your clinical assessment of a stroke patient include? (you may select more than one option)

- ☐ Visual acuity
- ☐ Refraction
- ☐ Visual field assessment
- ☐ Visual perceptual (e.g. visual neglect, visual agnosia)
- ☐ Ocular alignment
- ☐ Ocular movements
- ☐ Binocular vision
- ☐ Contrast sensitivity
- ☐ Colour Vision
- ☐ Lid assessment
- ☐ Pupil assessment
- ☐ Functional vision assessment
- ☐ Driving assessment
- ☐ Other

Please specify "Other" and any specific tool(s) used for these assessments

____

What test(s) do you perform for a visual acuity assessment? (you may select more than one option)

- ☐ LogMAR Chart
- ☐ Snellen Chart
- ☐ Gratings (e.g. Lea Paddles, TAC)
- ☐ Near visual acuity
- ☐ Fixing and following
- ☐ Other

Please specify "Other" ________________________________

What test(s) do you perform for refraction? (you may select more than one option)

- ☐ Subjective refraction
- ☐ Objective refraction (retinoscopy)
- ☐ Other

Please specify "Other" ________________________________

What test(s) do you perform for a visual field assessment? (you may select more than one option)

- ☐ Confrontation
- ☐ Goldmann perimetry
- ☐ Automated perimetry
- ☐ Bjerrum tangent screen
- ☐ Other

Please specify "Other" ________________________________

What test(s) do you perform for an assessment of ocular alignment? (you may select more than one option)

- ☐ Assessment of corneal reflections

- ☐ Cover test
- ☐ Other

Please specify "Other" ____________________

What test(s) do you perform for an assessment of ocular movements? (you may select more than one option)

- ☐ Nine positions of gaze
- ☐ Convergence
- ☐ Hess chart
- ☐ Saccades
- ☐ Smooth pursuit
- ☐ Vestibular ocular reflex
- ☐ Optokinetic Nystagmus (OKN)
- ☐ Other

Please specify "Other" ____________________

What test(s) do you perform for an assessment of binocular vision? (you may select more than one option)

- ☐ Lang I or II
- ☐ TNO
- ☐ Timtus
- ☐ Randot
- ☐ Frisby Davis (near test)
- ☐ Frisby Davis 2 (distance test)
- ☐ Prism fusion range
- ☐ 20 dioptre prism fusion test
- ☐ Bagolini striated glasses test
- ☐ Worth 4 light test
- ☐ Synoptophore investigation
- ☐ Other

Please specify "Other" ____________________

What test(s) do you perform for an assessment of visual perception? (you may select more than one option)

- ☐ Line bisection tests
- ☐ Cancelation tasks
- ☐ Copying
- ☐ Drawing
- ☐ Other

Please specify "Other" ____________________

What test(s) do you perform for an assessment of contrast sensitivity? (you may select more than one option)

- ☐ Pelli-Robson
- ☐ Vistech
- ☐ Melbourne Edge Test
- ☐ Other

Please specify "Other" ________________________

What test(s) do you perform for an assessment of colour vision? (you may select more than one option)

□ Ishihara

□ CUVCT

□ Farnsworth Munsell D-15

□ Farnsworth Munsell 100 Hue Test

□ Other

Please specify "Other" ________________________

What test(s) do you perform for an assessment of functional vision? (you may select more than one option)

□ Observations of function/navigation

□ Spatial awareness

□ Eye scanning

□ Reading

□ Writing

□ Activities of daily living

□ Mobility

□ A validated visual function questionnaire

□ Locally developed visual function questionnaire

□ Other

Please specify which questionnaire/tool you use: ____________

Please specify "Other" ________________________

Visual Rehabilitation

What symptoms do patients present with in your clinic? (you may select more than one option)

- ☐ Loss of peripheral vision or parts of vision
- ☐ Blurred vision
- ☐ Shaky vision
- ☐ Diplopia
- ☐ Abnormal head posture
- ☐ Depth perception difficulty
- ☐ Bumping into things/poor spatial awareness
- ☐ Recognition difficulties
- ☐ Reading difficulty
- ☐ Writing difficulty
- ☐ Lid droop
- ☐ Facial droop
- ☐ Photophobia
- ☐ Colour vision changes
- ☐ Other

Please specify "Other" ________________________________

Which post-stroke visual conditions do you see in your clinic? (you may select more than one option)

- ☐ Visual field loss
- ☐ Reduced visual acuity
- ☐ Reduced contrast sensitivity
- ☐ Strabismus
- ☐ Eye-tracking defect
- ☐ Ocular gaze palsy
- ☐ Nystagmus
- ☐ Perceptual defect (e.g. neglect, agnosia)
- ☐ Lid defect
- ☐ Pupil defect
- ☐ Colour defect
- ☐ Other

Please specify "Other" ________________________________

What treatment/therapy modalities do you prescribe? (you may select more than one)

- ☐ Prisms for diplopia
- ☐ Prisms for visual field loss
- ☐ Prisms for gaze
- ☐ Monocular patch for diplopia
- ☐ Compensatory head posture for diplopia
- ☐ Optical correction (prescription glasses)
- ☐ Reading strategies

☐ Visual scanning for visual field loss

☐ Visual scanning for gaze

☐ Low vision aids (e.g. magnifiers)

☐ Visual aids (e.g. rulers, typoscopes)

☐ Orthoptic exercises (e.g. convergence exercises)

☐ Mirror therapy

☐ Vision related advice

☐ Functional advice

☐ Other

Please specify "Other" ______________________________

Do you refer to, or work with, an ophthalmologist for the medical management of post-stroke vision difficulties? (e.g. surgical intervention)

○ Yes

○ No

Which medical management options are ophthalmologists consulted for? (you may select more than one)

☐ Surgical intervention

☐ Botulinum toxin

☐ Pharmacological treatment

☐ Other

Please specify "Other" ________________________________

Typically, when do you discharge or cease the follow up of patients with post-stroke difficulties? (you may select more than one)

☐ After the first consultation

☐ Within 1 month

☐ Within 2 - 3 months

☐ Within 3 - 6 months

☐ Within 6 - 9 months

☐ Within 9 -12 months

☐ After 12 months

☐ Review indefinitely

Do you collaborate with other health professionals in delivering post-stroke rehabilitation services?

○ Yes

○ No

Which health professionals do you typically collaborate with in the delivery of rehabilitation services? (you may select more than one)

☐ Occupational therapists

☐ Speech pathologists

☐ Physiotherapists

☐ Psychologists

☐ Social workers

☐ Neurologists

☐ Ophthalmologists

☐ Optometrists

☐ Other

Please specify "Other" ________________________________

Service Enablers and Barriers

How would you rate your confidence in assessing a patient with post-stroke vision difficulties?

○ Extremely confident

- ○ Highly confident
- ○ Confident
- ○ Slightly confident
- ○ Not at all confident

How would you rate your confidence in managing a patient with post-stroke vision difficulties?

- ○ Extremely confident
- ○ Highly confident
- ○ Confident
- ○ Slightly confident
- ○ Not at all confident

How would you rate your confidence in adapting your approach for patients with communication difficulties when delivering post-stroke vision services?

- ○ Extremely confident
- ○ Highly confident
- ○ Confident
- ○ Slightly confident
- ○ Not at all confident

How would you rate the effectiveness of the collaboration or multi-disciplinary team you work with?

- ○ Excellent
- ○ Good
- ○ Fair
- ○ Poor
- ○ Very Poor

What do you perceive as the barriers for stroke patients receiving post-stroke orthoptic services? (you may select more than one option)

- □ Lack of referrals from other health professionals
- □ Lack of knowledge of the role of the orthoptist in stroke care
- □ Vision not prioritised in patient goal setting
- □ Limited orthoptists with skills or experience in stroke care
- □ Limited time available in clinic to provide comprehensive stroke care
- □ Absence of orthoptists in multidisciplinary stroke allied health teams
- □ Absence of orthoptics from the Stroke Foundation Clinical Guidelines for Stroke Management
- □ Patient access to care
- □ Financial considerations
- □ There are no barriers to care
- □ Other

Please specify "Other" ____

Are there specific resources, policies, or protocols that you perceive as enabling the provision of orthoptic services to stroke survivors?

____

What improvements or initiatives do you think could enhance access to orthoptists for post-stroke vision rehabilitation services or improve the provision of post-stroke orthoptic services.

____

Please provide any additional comments regarding post-stroke vision care services that you feel are important but were not addressed in this survey.

____
